# Quality and safety: Precision, accuracy and compliance with accepted standards of care

**DOI:** 10.4102/phcfm.v2i1.245

**Published:** 2010-12-01

**Authors:** Susara J. Oosthuizen, Claire Van Deventer

**Affiliations:** 1 Tshwane/Metsweding Region, Gauteng Department of Health, South Africa; 2 Department of Family Medicine, University of the Witwatersrand, South Africa

*Practise and prescribe to the best of your ability and judgement for the good of the patient, and never do harm.* −Hippocrates

An understanding of the concepts and application of quality care and quality assurance is fundamental to improving primary health care. The World Health Organization and the Millennium Development Goals both emphasise the need for cost-effective and safe health care for all.^1^ The deficiencies in the quality and safety of care delivered by health care institutions worldwide, especially in the developing world, are of great concern to administrators and health care users. Health care providers have become alarmed at statistics such as the reported deaths of 44 000–98 000 hospitalised patients annually in the United States of America, after negligent care or medical error.^2,3^ Ineffective care, injury to a patient or an adverse event not only result in expensive litigation but also in the silent complications of sub-optimal care.

Noting the lack of data on quality, some health care providers have revised their strategies to include performance indicators for quality, safety and other measurable outcomes of care. A study of the effects of accreditation on the quality of hospital care in Kwa-Zulu Natal showed that, although hospital compliance with accreditation standards improved, the quality of care in resource-constrained public hospitals did not necessarily improve.^4^ Whilst individual health carers are under the impression that they achieve high quality and safe care, errors occur because of a lack of knowledge, inadequate procedural skills and poor judgement.^5^ Deviations from accepted standards of care occur not only as a result of human error, but also because of systemic problems such as a lack of access, inequity, poor infrastructure and long waiting times. Deficiencies in care are generally not measured under normative standards.^6^

The physician-patient relationship and patient centeredness are therefore very important in quality care improvement programmes.^8^ The involvement of patients in the management of adverse events and active quality improvement projects need to be considered.

Despite all the evidence that quality care is not happening and that sub-optimal care is a significant cause of morbidity and mortality, the indicators to measure care are vague and the attainment of outcomes such as the Millennium Development Goals is in jeopardy. Health care managers need new indicators and interventions to monitor and improve the quality of care. These need not be technologically complex or expensive: for example, the application of information technology has demonstrated only marginally better outcomes with more or less the same number of adverse events.^9^

We should rather look at the quality of the teams providing health care. The building up of skilled teams and the retention of trained staff is crucial to quality service delivery. Team members should function with a shared understanding of what a quality service entails. The members need to communicate effectively and must be able to adapt quickly to changes in the environment. Optimal teamwork does not happen automatically, so interdisciplinary team training is essential if we are to provide consistent, quality health care.^2^ Dissatisfied staff and burnout contribute to the breakdown of the physician-patient relationship, poor adherence to treatment regimens and, inevitably, to adverse events. In some countries, health care managers use performance evaluations, audits and reward systems to correct this situation.

Clinical audits in daily practice are a vital link between health care and research. The information obtained from such audits can assist in identifying clinical workload and overuse of facilities. Many tools already exist which could be used to monitor practice and clinical and financial outcomes.^10,11,12,13^ Health administrators can use information about overload, clinical workflow, patient engagement and other systems problems, to bring about an improvement in the systems and care provided.

Practitioners need to understand the importance of adherence to protocols and national guidelines: precision and accuracy should be the norm when dealing with patients. All health care providers should train teams of health care workers in service, technology, procedural skills and medicine, with an emphasis on efficiency. Health authorities should restructure systems to deliver health services that provide for people’s needs and expectations and culminate in better outcomes.

**TABLE 1:**
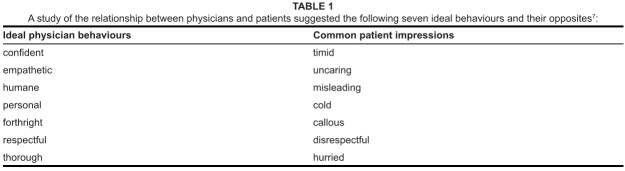
A study of the relationship between physicians and patients suggested the following seven ideal behaviours and their opposites^7^

In the more developed countries there is a trend towards upscaling quality improvement programmes to benefit millions of people.^14,15,16^ The local context and sustainability in developing countries should however always be considered when projects are developed.

## Key questions

If accreditation does not ensure quality of care − what would?
How do we get to precision, accuracy and compliance with accepted standards of care in our context?
How does one recognise suboptimal quality and/or early safety threats in one’s practice?
How does one overcome resource constraints in the application of quality care?
Where could one benchmark excellent care?
